# Deep brain stimulation in PD: risk of complications, morbidity, and hospitalizations: a systematic review

**DOI:** 10.3389/fnagi.2023.1258190

**Published:** 2023-11-17

**Authors:** Markey C. Olson, Holly Shill, Francisco Ponce, Sana Aslam

**Affiliations:** ^1^Department of Neurology, Muhammad Ali Movement Disorders Clinic, Barrow Neurological Institute, St Joseph’s Hospital and Medical Center, Phoenix, AZ, United States; ^2^Department of Neurosurgery, Barrow Brain and Spine, Barrow Neurological Institute, St Joseph’s Hospital and Medical Center, Phoenix, AZ, United States

**Keywords:** Parkinson’s disease, hospitalization, deep brain stimulation, DBS complications, DBS outcomes

## Abstract

**Introduction:**

Parkinson’s disease (PD) is a progressive and debilitating neurological disorder. While dopaminergic medication improves PD symptoms, continued management is complicated by continued symptom progression, increasing medication fluctuations, and medication-related dyskinesia. Deep brain stimulation (DBS) surgery is a well-accepted and widespread treatment often utilized to address these symptoms in advanced PD. However, DBS may also lead to complications requiring hospitalization. In addition, patients with PD and DBS may have specialized care needs during hospitalization.

**Methods:**

This systematic review seeks to characterize the complications and risk of hospitalization following DBS surgery. Patient risk factors and modifications to DBS surgical techniques that may affect surgical risk are also discussed.

**Results:**

It is found that, when candidates are carefully screened, DBS is a relatively low-risk procedure, but rate of hospitalization is somewhat increased for DBS patients.

**Discussion:**

More research is needed to determine the relative influence of more advanced disease vs. DBS itself in increased rate of hospitalization, but education about DBS and PD is important to insure effective patient care within the hospital.

## 1. Introduction

Parkinson’s disease (PD) is a neurodegenerative disorder characterized by progressive motor and non-motor symptoms. The cardinal symptoms of the disease are tremor, bradykinesia, rigidity, and postural instability and gait dysfunction, most of which are initially well-controlled with the use of carbidopa-levodopa and other pharmacological options. However, as PD progresses, the therapeutic window narrows, leading to both wearing off and medication-related dyskinesias (Jankovic, 2005; Vijiaratnam and Foltynie, 2020; Teymourian et al., 2022). Many patients experiencing these symptoms decide to pursue surgical options, which can provide a “second honey-moon” period that restores much of the symptom relief initially experienced when they began medication (Simonin et al., 2009).

Deep brain stimulation (DBS) is one well-established surgical technique for PD (Krack et al., 2019). While it is not understood precisely how DBS affects the dopaminergic networks within the brain leading to response, drastic symptomatic improvement is noted for many patients. Due to its relative safety, customizability, and reversibility, DBS has supplanted previous lesioning techniques as standard-of-care since its approval for PD in 2002 in the US (Yu and Neimat, 2008; Collins et al., 2010). The use of DBS has increased over time as well, with recent estimates suggesting that 244,000 devices have been implanted globally (Wong et al., 2023).

However, DBS is not entirely without risk, and complications such as infection, hemorrhage, and even mortality have been noted. Post-operative side effects such as confusion, delirium, and cognitive decline have also been found. Any of these conditions may lead to hospitalization following DBS. Some symptoms of PD that lead to increased risks and may require specialized care within the hospitalized setting, such as dysphagia and falls, may also not be well-addressed by DBS (Nantel et al., 2012; Troche et al., 2013; Crispo et al., 2021). Given that DBS is most often used in the later stages of PD, both continuing symptom progression and complications from DBS itself may play a role in increasing rates of hospitalization.

In addition to experiencing higher rates of hospitalization, patients with PD experience increased complications during hospitalizations (Shahgholi et al., 2017). The risk of falls, aspiration, and delirium are higher than age matched controls (Martignoni et al., 2004; Aminoff et al., 2011), which increases risk of injury, pneumonia, and other adverse outcomes. Due to high patient loads and lack of knowledge among hospital personnel about the importance of timing in levodopa response, many patients also experience missed or delayed medication doses in the hospital, further increasing complications, as advanced PD patients are often on complicated and time sensitive regimens (Hou et al., 2012).

It is unclear what role, if any, DBS plays in affecting outcomes in hospitalized PD patients, be it related to DBS surgery or not. The goal of this systematic review is to better understand the body of literature in regard to risks associated with DBS in both the short- and long-term, as well as to characterize the effects of both preoperative patient and surgical factors on risk in DBS. We hope to use this knowledge both to encourage further study regarding the needs of patients with PD and DBS to prevent poor outcomes and to guide education in the treatment of individuals with PD and DBS within the hospital setting.

## 2. Methods

**FIGURE 1:**
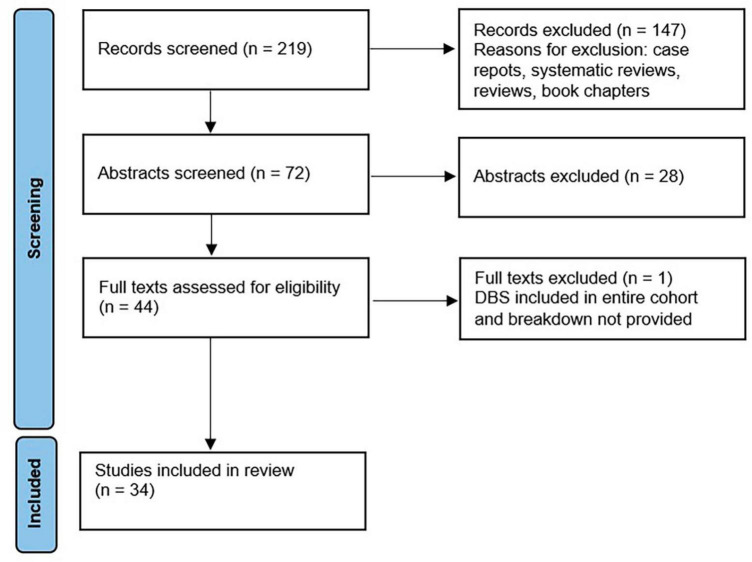
PRISMA diagram of review process.

Literature search of PubMed/MEDLINE was conducted in March 2023, according to PRISMA guidelines. Search terms included {[parkinson*(Title/Abstract)] AND [deep brain stimulation (Title/Abstract) OR dbs (Title/Abstract)]} AND [hospital*(Title/Abstract)], which generated 219 results. Only English-language original trials that discussed rate of hospitalization and/or complications following DBS surgery were included. Review articles were not included, but were reviewed for potentially missed articles. Following separate title and abstract review by authors S.A. and M.C.O. (detailed in Figure 1), both authors compared results and came to a consensus on papers in which disagreement had occurred. Papers in which both authors came to an approval decision were included in full text review. Publications were categorized as related to surgical procedure, short term outcomes defined as outcomes measured perioperatively and within 90 days of surgery, and long term outcomes defined as measures assessed greater than 90 days of DBS surgery.

## 3. Patient risk factors for surgical complication

While DBS has proven to be a safe, well-tolerated, and beneficial intervention for a large number of patients, not all patients demonstrate the same hopeful outcomes, and outcome is likely to be affected by certain patient characteristics. Patients with PD in particular may be at increased risk of readmission and revision relative to patients with essential tremor, dystonia, or epilepsy (Tafreshi et al., 2021). Among patients with PD, multiple papers suggest that older age, lower preoperative cognitive ability, and comorbidities such as coronary artery disease and obesity negatively affect postoperative outcomes, increasing risk of delirium and lengthened hospital stay. Patients with greater number of comorbidities were at higher risk of complication, though comorbidities were not categorized or measured in a standardized fashion across studies (Mikos et al., 2010; Rughani et al., 2013; Rumalla et al., 2018; Young et al., 2019; Lu et al., 2022; Zhang et al., 2022).

However, at least for age, this consensus is not universal, with individual papers finding no association between age and post-operative confusion (Abboud et al., 2019) or 90-day complication rates (DeLong et al., 2014). “Older age” was also variably characterized between studies, and those that found associations between age and increased complications may have been comparing older individuals with greater comorbidities in comparison with those that did not. To address this potential confound, DeLong et al. (2014) specifically investigated the effect of age on DBS outcomes, utilizing epochs of 5 years and ranging from <50 to >90 years in a cohort from a national database with a sample size of over 1,700 patients. They defined “older age” as >75 years and did not find any difference in outcomes. Thus, age may be less of a factor in outcomes than the presence of cognitive decline or other health issues.

Certain kinds of cognitive dysfunction may also be more indicative of need for caution proceeding to DBS than others. Abboud et al. (2015) also indicates that, while impairments in attention and visuospatial processing negatively affect postoperative outcomes, a diagnosis of mild cognitive impairment (MCI) alone does not. It was also suggested that premorbid PD severity and phenotype may play a role in risk for complications, with Unified Parkinson’s Disease Rating Scale (UPDRS) part III motor scores directly correlated with rate of postoperative confusion. Noted imbalance and history of falls prior to surgery also were associated with confusion (Abboud et al., 2019).

This research indicates that, by reviewing patients carefully to determine those that are likely to be safe candidates for surgery, postoperative outcomes can be improved, and complications reduced. Many neurological centers, including that of the authors, have taken these conclusions into account and carefully weigh patients’ ability to proceed to DBS based on physical and neuropsychological testing, symptom phenotype, responsiveness of symptoms (as measured by UPDRS Part III scores) to levodopa in off-on testing, and presence of wearing off or medication-induced dyskinesia. To take all of these factors into consideration, consensus panels are formed in which individuals from neurology, neurosurgery, neuropsychology, and often neurorehabilitation (physical, occupational, and/or speech therapy) meet together to discuss potential DBS candidates on a regular basis. An article by Higuchi et al. (2016), which found that patients identified during interdisciplinary evaluation for DBS that were marked with major or minor concerns from any specialty service had more unintended hospitalizations. However, this kind of committee may not be feasible always, especially at smaller institutions.

Regional trends, insurance types, and hospital factors were reviewed by Sharma et al. (2013) and found that patients were more likely to experience complications if they were elderly, female, had non-private insurance, reported higher number of comorbidities, or were undergoing surgery by surgeons with low annual caseload or at low volume centers. Surgical centers with higher volumes demonstrated significantly lower side effect profiles in several other studies as well (Eskandar et al., 2003; Kalakoti et al., 2015); however, this effect is not unchallenged, with one large study actually showing a non-significant trend toward lower complication rates in smaller centers (McGovern et al., 2013). All three of these studies involved data from thousands of patients, so it is unlikely that the difference in effects was due to variability or small sample size, but may reflect differences in reporting/measurement of adverse outcomes or categorization of centers by size, or differing practices or level of ability within the smaller centers analyzed within McGovern et al. (2013). It would appear that at least some small centers have thus far struggled with higher complication rates than those experienced by large centers, which may benefit from more clear patient selection methods as well as more experienced surgeons and post-operative staff and increased access to the newest technology and methods.

## 4. Short-term complications of DBS surgery

The majority of complications from DBS requiring hospitalization are expected to take place in the duration immediately after implantation and may include standard surgical risks such as bleeding, inflammation, infection, and reactions to anesthesia or other medications as well as device-specific problems such as lead malfunction and migration. Other problematic effects, such as cognitive decline, are harder to attribute fully to either surgery or device-related symptoms but have a large impact on the care required by patients in the hospital.

Post-operative delirium, especially, contributes to poorer functional outcomes, higher risk of institutionalization, and mortality, and these effects are hard to combat once delirium has set in Rieck et al. (1995). There is conflicting data on whether the preventative use of antipsychotics may reduce rates of delirium (Neufeld et al., 2016; Janssen et al., 2019), but most antipsychotics are contraindicated in PD due to their likelihood of worsening motor symptoms. Management of delirium in PD should thus generally be restricted to management of precipitating factors (such as infection and pain), reduction of medications that may be exacerbating delirium, and provision of environmental support (Ebersbach et al., 2019).

To better understand the specific complications PD patients experience in the period shortly after surgery, and recommend care that may reduce negative effects associated with these complications within the hospital, we specifically looked at those papers discussing the first 90 days post-operatively. Fifteen papers met these criteria (Table 1). Sample sizes ranged from 27 to 6,058.

**TABLE 1:** Publications reporting short term outcomes following DBS surgery (3 months or less).

References	Patient population	Outcomes duration	Complications reported/assessed	Risk factors noted
Abboud et al., 2015 *	PD DBS = 130	Immediate post-op	Postoperative confusion and hospitalization days	MCI presence, type, and DRS performance did not affect immediate outcomes. Attention impairment predicted longer postoperative hospitalization and showed a trend toward occurrence of postoperative confusion.
Abboud et al., 2019	PD DBS = 130 (48 with tremor-predominant)	Immediate post-op	7 cases of postoperative confusion and 19 of prolonged postoperative hospitalization (>2 days).	Non-tremor predominant phenotype 10.1% had confusion; preoperative falls/balance-dysfunction. Each point increase in UPDRS III/MDS-UPDRS III score, the odds of having postoperative confusion increased by 10%
Carlson et al., 2014	PD DBS = 59	Immediate post-op	Delirium following DBS leads 22%, following generator placement 10%	History of delirium, age, and disease duration, opiate equivalent doses, missed and delayed PD medication doses
DeLong et al., 2014	PD DBS = 1,757	90 days	7.5% experienced at least 1 complication: wound infections (3.6%), pneumonia (2.3%), hemorrhage or hematoma (1.4%), or PE (0.6%).	Age NOT correlated with increased complications
Eskandar et al., 2003	All surgical procedures = 1,650; PD DBS = 589		DBS inpatient mortality 0.2%.; Post-op complications 1.5% (0.5% hematomas, 1% mechanical, 0.3% removal.)	Hospitals with larger annual caseloads had lower mortality rate
Jiang et al., 2022 *	PD DBS = 27	Post-op	Postoperative complications 26.1% (most common: intracranial bleeding 6.1%, VH 21.7%)	
Kalakoti et al., 2015	PD DBS = 24,787	n/a	OR 2.88 unfavorable discharge, 2.48 cardiac complications, 2.51 increased LOS, 1.62 high end hospital charges.	Low-volume centers (<5 per year)
McGovern et al., 2013	PD DBS = 18,313	Post-op	13% “non-routine” discharges (5.39% home health care, 7.22% long-term care facility, 0.28% death). 6.49% perioperative complication.	Non-significant trend for the low-volume group to have lower complication rates (6.70% vs. 8.02%, *p* = 0.20), shorter LOS (2.12 v 2.35 days, *p* = 0.06), and lower mortality (0.12 vs. 0.30, *p* = 0.15), but also lower routine discharges (85.31% vs. 88.75%, *p* = 0.15). Comorbidity score associated with perioperative complications.
Ramayya et al., 2017	Total DBS = 347, PD DBS = 235	30 days	30 day readmission 6.6% (3.7% surgery related complications).	Increasing age
Rughani et al., 2013	Total = 5,464 patients (PD = 4,145, DBS = 4,961; DBS for PD = 3,762)	Immediate post-op	In-hospital mortality was 0.26% (0.15% related to surgical factors.) PD patients were more likely to suffer hemorrhage or stroke. PD post-DBS mortality rate was 0.32%.	Increasing age, medical comorbidities. Age >70 years, the risk of in-hospital death was 0.42%, the risk of any complication was 3.55%, and the risk of hemorrhage or stroke was 1.93%
Rumalla et al., 2018	DBS = 3,392 patients (PD 70.7%, ET 25.6%, dystonia 3.7%)	30 and 90 days	30 day PD readmission 2.1% and 90 day PD readmission 4.8%	PD 30 days RR: Age 75 + 4.5%, coronary artery disease 6.0%, obesity 6.3%, HLD 3%. PD 90 days RR: Age 75 + 7.9%, coronary artery disease 11.4%, obesity 14%, HLD 5.7%
Schneider et al., 2020	PD DBS = 6,058	30 days	Non-elective readmission 4.9%	Socioeconomic status, comorbidity burden, and teaching hospital status.
Strapasson et al., 2019	PD with STN DBS = 49	24 h	Post-op confusion incidence 26.5%	Charleson comorbidity index > 2
Tafreshi et al., 2021 *	PD DBS = 3,230	1 month	30-day readmission rate 15.5% - causes of readmission: pneumonia 1.6%, dysphagia 0%, DBS revision 11.5%.	
Young et al., 2019	PD DBS = 183	30 days	29% had length of stay >2 days	Age > 70, frequent falls, poor social support

DBS, deep brain stimulation; DRS, dementia rating scale; LOS, length of stay; MCI, mild cognitive impairment; PE, pulmonary embolism; PD, Parkinson’s disease; RR, relative risk; STN, subthalamic nucleus; VH, visual hallucinations. *Publications which reported both short and longterm outcomes.

Six papers focused on immediate post-operative outcomes, mainly postoperative delirium. Rates of postoperative delirium ranged from 5.4 to 26.5%, with smaller studies reporting greater incidence (Carlson et al., 2014; Abboud et al., 2015, 2019; Strapasson et al., 2019). The wide range of risk reported may be due in part to surgical centers with higher volumes demonstrating lower side effect profiles in most studies (Eskandar et al., 2003; Kalakoti et al., 2015). While delirium poses a significant risk, as discussed above, most cases of delirium following DBS resolved following the immediate postoperative period and rarely resulted in permanent neurological deficits. This may indicate that centers performing DBS have specifically trained their personnel regarding the needs of individuals with PD during episodes of delirium or instituted practices to ensure contraindicated medications are not given. However, two papers evaluating discharge dispositions found that unfavorable discharges, i.e., to a facility other than home, ranged from 5.9 to 13% following DBS surgery (McGovern et al., 2013; Kalakoti et al., 2015), indicating that there is still much room to research methods of improving patient outcomes and institute these methods within hospital settings.

Also analyzed immediately postoperatively were in-hospital mortality following DBS surgery, which was 0.26% overall (0.15% related to surgery itself) and 0.032% for individuals with PD (Rughani et al., 2013), and perioperative complications, which were reported at 6.49% (McGovern et al., 2013). Young et al. (2019) reported that 29% of their cohort had longer than a 2-day hospital stay following surgery. Eskandar et al. (2003) did not specify postoperative follow up timeline, but found similar mortality rates (0.2%), with postoperative complications affecting 1.5% of DBS patients. Of these, 0.5% reported hematomas and 1% mechanical problems, and 0.3% of cases required explant of the device due to these complications.

Age and/or other preoperative risk factors/comorbidities also likely play a large role in the disparities in complication rates reported across studies. Postoperative complications in individuals aged 75 years or older may be as high as 26.1%, with 0.5% of patients experiencing hematomas, 1% having mechanical difficulties, 0.3% of implants requiring removal (Jiang et al., 2022). This rate is significantly higher than those reported in other studies, which is consistent with the finding that age is a risk factor to be taken into consideration in surgical candidacy decisions, though it may be modulated by presence or absence of other risk factors such as comorbidities. However, it should be noted that the benefit of DBS in terms of improvement in motor outcomes did not seem to be lower for older patients (DeLong et al., 2014). Given the severity of the complications for the 26.1% of older patients experiencing them in Jiang et al. (2022) was not given, and rate of required explant, mechanical problem, and hematoma were the same as those given for patients on average in Eskandar et al. (2003), some older patients, especially those with very severe tremor, dyskinesia, or medication fluctuations, may be willing to accept the greater risk. Careful consideration of risk-benefit for DBS, both with the patient and in a consensus review, should be undertaken in these cases. Alternative therapies such as focused ultrasound may also be considered, but the unilateral nature of FUS may make it infeasible for individuals with traditional, bilateral disease progression.

While the immediate post-operative period may present the most concern for severe complications, readmissions weeks to months after surgery do occur. Four studies reported on DBS safety based on 30 days of follow-up after surgery, focusing mainly on readmissions to the hospital. Hospital readmissions ranged from 2.1 to 15.5% (Ramayya et al., 2017; Rumalla et al., 2018; Schneider et al., 2020; Tafreshi et al., 2021). However, these numbers reflect total readmissions during the follow-up period, and may be associated with progression of PD symptoms or symptoms of comorbidities and thus unrelated to DBS. Ramayya et al. (2017) found admissions directly related to DBS surgery/complications to be 56% of total admissions (3.7% of patients were admitted due to surgical complication while 6.6% were admitted to the hospital for any reason in the 30-day postoperative period). In contrast, Tafreshi et al. (2021) found that 11.5% of patients were readmitted for DBS revision.

Two studies looked at effects of DBS on hospitalization and complication rates at 90 days postoperatively. Rumalla et al. (2018) found that 4.8% of DBS patients were readmitted to the hospital within 3 months of DBS surgery, which is more than double the 30-day postoperative readmission rate of 2.1% reported within the same paper. This may be due to the natural rate of hospitalization within PD populations over time, especially those with advanced disease more likely to receive DBS. However, DeLong et al. (2014) did find that 7.5% of patients in their large sample of 1,757 PD individuals suffered from at least one postoperative complication within the 90-day postoperative window, including wound infections (3.6%), pneumonia (2.3%), hemorrhage or hematoma (1.4%), or PE (0.6%). Both studies were large retrospective reviews of national databases: DeLong et al. (2014) analyzed the Thomson Reuters MarketScan national database that examined 1,757 patients who underwent DBS for PD during the period from 2000 to 2009, while Rumalla et al. (2018) looked at records from the US Nationwide Readmissions Database for 3,392 DBS patients (2,398 with PD). Given the significant number of patients that appear to experience later surgery-related complications, it is important that patients continue to be followed regularly by their neurologist within the post-operative period, even upon completion of DBS programming. It is also urgent that, upon hospitalization for an individual with recent DBS, the patient’s neurologist or neurosurgeon be notified to advise care.

## 5. Long-term outcomes of DBS

As patients get further out from surgery, it may become more difficult to determine the precise effect of DBS vs. disease progression on complication and hospitalization, barring clear diagnosis such as lead migration/revision or device-related infection. However, even in the case of ambiguous hospitalization, care needs may be affected by presence of DBS.

Eleven papers analyzed the effects of DBS on complication rates in the long-term (Table 2). Sample sizes ranged from 27 subjects with PD and DBS and single center to 32,988 subjects from CMS databases. Some groups only reported DBS data whereas others primarily evaluated PD and included breakdown of data for those with DBS within the cohort. The wide variety of study types, analysis methods, and definitions for complications makes it difficult to directly compare studies, but some information can still be gleaned.

**TABLE 2:** Publications reporting longterm outcomes following DBS surgery (greater than 3 months).

References	Patient population	Outcomes duration	Complications reported/assessed	Risk factors noted
Abboud et al., 2015	PD DBS = 56	6 months and 1 year	Cognitive dysfunction	Visuospatial impairment showed trend toward less improvement in 6-month functional score and 1-year QOL
Chen et al., 2022	PD DBS = 211 (191 no infection group, 20 infection group)	6 months	DBS related infections	BMI, blood glucose, albumin
Deng et al., 2020	Total = 44,866, PD DBS = 32,988	n/a	Complications 4.4% (highest was 1.8% urinary/renal), in-hospital mortality 0.2%	Medicaid patients higher risk than Medicare, APR-DRG illness classification and preop mortality risk.
Haas et al., 2019	PD DBS = 25	6–15 months	Most common persistent complications: “incitement disorders (i.e., apathy)” and “falls” (each 11.9). “Lead revision” and “apraxia of eyelid opening” (1.7%) rarely occurred within the QualiPa registry	
Hassan et al., 2013	Total PD = 3,415 (1,120 had hospital encounter at 1 year follow-up); PD DBS = 356	1 year	Rate of hospitalization (48%, vs. those without DBS 31%)	Not entirely stratified by DBS; overall, risk of 1+ encounters: more severe PD, motor fluctuations, prolonged TUG. New presentation to hospital: advanced disease, comorbidities, DBS, cognitive impairment, and female gender.
Higuchi et al., 2016	Total PD = 164, PD DBS = 133	1 year	21% experienced an unintended hospitalization	Concerns from any specialty service during interdisciplinary evaluation: major concern 89%, minor 33%, none 3% hospitalization rate. Strong relationship between worsened PDQ-39 at 12 months and increased hospitalization.
Jiang et al., 2022	PD DBS = 27	55 months (mean)	Exposed wire at 4 months 4.3%	
Kortz et al., 2021	Total = 27,956, PD DBS = 18,883	n/a	Complications 4.5%, long LOS 1.3%, negative disposition 11.2%, high charge 2.9%	Neuropsychiatric comorbidity was a significant independent predictor of unfavorable outcome, with the greatest impact on LOS and complication risk
Scelzo et al., 2019	Total PD = 182, PD DBS = 91	1 year	Risk of hospital admissions related to PD was similar when excluding DBS related admissions. Risk of death and dementia was similar.	
Shahgholi et al., 2017	Total = 7,507 (did not report DBS outcomes separately)	5 years	25.6% had a history of a hospital encounter prior to baseline.	Hospital encounter prior to baseline: race (white race: OR 0.49), utilization of physical therapy, DBS, # of comorbidities, caregiver strain, and TUG. Time to hospital encounter from baseline associated with age and number of medications. Time to a second hospital encounter associated with caregiver strain and number of comorbidities.
Sharma et al., 2013	Total PD = 14,291, PD DBS = 2,228	n/a	Inpatient mortality 0.17%, one or more complications 1.02%	Elderly female patients with non-private insurance and high comorbidity index who underwent surgery at low-volume centers performed by a surgeon with a low annual case volume and the occurrence of postoperative complications were correlated with an adverse discharge disposition
Tafreshi et al., 2021	PD DBS = 3,230	3 months and 6 months	3 months: readmission 23.7%, dysphagia 0%, DBS revision 9.8%; 6 months: readmission 29.8%, dysphagia 0%, DBS revision 8.6%	

APR-DRG, All Patients Refined Diagnosis Related Groups; DBS, deep brain stimulation; DRS, dementia rating scale; LOS, length of stay; MCI, mild cognitive impairment; n/a, not applicable; OR, odds ratio; PD, Parkinson’s disease; QOL, quality of life; RR, relative risk; STN, subthalamic nucleus; TUG, timed up and go test.

Four papers collected information about complications during 6 months of follow-up. Incidence of cognitive dysfunction and DBS-related infections was noted, but precise rates of occurrence were not recorded (Abboud et al., 2015; Chen et al., 2022). Other complications included apathy and falls (11.9% incidence each) and apraxia of eyelid opening (1.7%) (Haas et al., 2019). With the exception of DBS infection, it is likely that typical PD progression played a large role in the incidence of these complications. However, regardless of cause, apathy and falls hugely impact hospital care.

Tafreshi et al. (2021) reported readmission rates of 29.8% within 6 months of surgery, with 8.6% of patients requiring lead revision, but much lower rates of revision (1.7%) were reported by Haas et al. (2019), though this was a pilot study analysis with only 25 patients. The former analysis was based on a queried data from The National Readmission Database with 3,230 PD DBS patients. In the case of lead revision, it is expected that patients likely returned to the hospital of their initial DBS implantation, making patient records and physicians easily accessible, but in the case of hospital or provider switch, it is key that existing records be reviewed carefully to best guide surgical revision plan and post-operative care.

Four articles followed patient outcomes for 1 year post-DBS. Abboud et al. (2015) reported continued cognitive dysfunction 1 year postoperatively. The other three papers focused on rates of hospitalization, with two comparing rates of hospitalization following DBS to that of non-surgical PD patients during the same period. However, both the rates of hospitalization (21–48%) and the conclusion whether DBS significantly increased hospitalizations differed between these articles (Hassan et al., 2013; Higuchi et al., 2016; Scelzo et al., 2019). Hassan et al. (2013) presented one of the only prospective studies of long-term comparative outcomes, comparing 356 patients with DBS and 3,415 PD patients total enrolled in the international multicenter National Parkinson’s Foundation (NPF) QII Study. However, this prospective follow-up was from the date of enrollment, not necessarily from the time of DBS surgery, increasing likelihood that hospitalizations are due to disease progression in the more advanced patients likely to have received DBS and not necessarily related to the DBS surgery itself. In this analysis, PD patients with DBS had a 48% rate of hospitalization compared to 31% of those without DBS. When Scelzo et al. (2019) excluded hospitalizations related specifically to DBS, they found a similar hospitalization rate, risk of death, and dementia in those with and without DBS in an observational study with 1 year followup.

Four additional studies reported retrospective data without a set followup timeline. Jiang et al. (2022) reported an exposed wire in one patient at 4 months postoperatively, but did not document other long-term complications. However, this was a small study (*N* = 27) specifically analyzing complications in a population of surgical patients that were 75 or older from a single institution. Three other large-scale retrospective chart reviews analyzing data from multiple institutions (populations of 2,228–32,988 patients following DBS for PD, with additional populations of either DBS for other disorders or patients with PD not receiving DBS for comparison) discussed long-term complication rates, which varied from 1.02 to 4.5%, and in-patient mortality rates of 0.17–0.2% (Sharma et al., 2013; Deng et al., 2020; Kortz et al., 2021). All of these studies indicate a relatively small, but certainly not insignificant, rate of complications following DBS and highlight a need for further research into the causes and potential methods of reduction to improve hospitalized patients’ outcomes and care following an adverse event.

## 6. Effects of different surgical approaches on DBS risk

While the papers discussed thus far have considered the risk profiles of DBS implantation as a whole, surgical techniques and technologies used actually vary considerably between centers, and even within surgeons at the same center. These differences may dramatically affect patient outcomes. However, there has been little systematic comparison of different surgical options. Nine papers discussed the effects of different procedural approaches to DBS and their effect on postoperative outcomes (Table 3), and most of these highlighted different approaches/technologies, making systematic analysis difficult.

**TABLE 3:** Publications related to surgery associated risk factors and aspects of DBS.

References	Patient population	Mean follow up	Procedure/intervention	Outcomes
Foltynie et al., 2010	PD = 79	12 months	No MER	Mean standard error in electrode placement 1.3 (0.6) mm. No hemorrhagic complications.
Sutcliffe et al., 2010	PD = 46 (LA = 20, GA = 26)		Local v. general anesthesia	Reduction in levodopa clinically and statistically significant for both: 6-month requirement LA 39.4% of preoperative, GA group 32.3%. Reduction in levodopa was maintained at 1 year. Mean duration of surgery was 8.2 h (7.8–8.6) for the LA group and 7.5 h (7.2–7.8) for the GA group (*p* = 0.003). Mean LOS was 5.4 days (4.6–6.3) for the LA group and 3.8 days (3.4–4.4) for the GA group (*p* = 0.001). No difference in electrophysiological recording.
Zahos and Shweikeh, 2013	Total = 18 (PD STN = 11)	8.2 months	Frameless surgery	No intraop complications. Two subjects (of total) developed wound dehiscence post-operatively and 1 had fall-induced lead fracture. Motor scores 58% reduction, levodopa usage 47%.
Ray et al., 2019	Total = 206 (PD = 156)		Transventricular approach	Complications 6.1% (highest were AMS/delirium 1.6%, ICH 1.2%, seizures 0.8%, death 0.8%)
Gologorsky et al., 2011	Total = 81 (145 leads)		Ventricular wall violation	9.9% postoperative confusion and increased LOS; all with complications had ventricular wall violation (8 of 16).
Lu et al., 2022	Total STN = 131	7 days	IV anesthesia	16.8% with postop delirium. Preop MMSE (OR 0.855) and UPDRS III “on” scores (OR 1.061) independently associated with postop delirium.
Goodman et al., 2006	Total = 100 (191 leads)	4 years		No deaths or permanent neurological deficits. Complications rate 37% of patients and 19% of implants (highest 5% device infection, postop confusion 6.8%, revisions 3.1%)
Jacob et al., 2016	Total = 211 (PD = 129)		Awake v. asleep cost	Asleep DBS cost $38,850 ± $4,830 was not significantly different than the awake DBS cost $40,052 ± $6,604. The standard deviation for asleep DBS was significantly lower. Readmission similar (awake 3.8%, asleep 4.4%).
Zhang et al., 2022	Total = 94 (PD = 93)	55.3 months		Overall extrusion rate 12.8% (6.4% at scalp, 6.4% at chest). Mean time from initial implantation to extrusion was 72.7 months for the scalp and 52.8 months for the chest.

DBS, deep brain stimulation; GA, general anesthesia; LA, local anesthesia; LOS, length of stay; PD, Parkinson’s disease; STN, subthalamic nucleus.

Two papers discussed the use of generalized vs. local anesthesia (asleep vs. awake DBS), finding that surgical outcome was similar between the groups in terms of symptomatic improvement, levodopa reduction and rate of readmissions, and that length of surgery and length of post-operative stay were actually reduced in the generalized anesthesia group, demonstrating the safety and efficacy of asleep DBS (Sutcliffe et al., 2010; Jacob et al., 2016). Lu et al. (2022) also examined the risks of delirium following asleep DBS, finding that 16.8% of patients experienced postoperative delirium, but did not directly compare to a group of patients undergoing DBS under only local anesthesia. Overall, asleep DBS seems to be safe and efficacious, and many patients may prefer that option or even experience stress at the thought of being alert during an operation, making the approach beneficial.

Additional papers analyzed the ability to perform DBS without traditionally used microelectrode recording (MER) for lead placement (Foltynie et al., 2010) or stereotactic frame for stabilization (Zahos and Shweikeh, 2013), finding efficacious placement with low rates of complications. However, neither paper compared results to that of a control group using traditional DBS lead placement procedures.

Another study using both MER and stereotactic frame found postoperative morbidity including 7 device infections (3.7%), 1 cerebral infarct, 1 intracerebral hematoma, 1 subdural hematoma, 1 air embolism, 2 wound hematomas requiring drainage (1.0%), 2 skin erosions over implanted hardware (1.0%), 3 periprocedural seizures (1.6%), 6 brain electrode revisions (3.1%), postoperative confusion in 13 patients (6.8%), and 16 battery failures (8.4%). Of the 100 patients (200 implants), there were no surgical deaths or permanent new neurological deficits. The average hospital stay for all 100 patients was 3.1 days (Goodman et al., 2006).

Two papers discussed the safety of a *trans-*ventricular approach for inserting DBS leads, with conflicting results regarding the relative safety profile of this approach. Ray et al. (2019) noted a low percentage of complications (6.1% of patients experiencing any complication, with 1.5% delirium, 1.2% hemorrhage, 0.8% seizures and 0.8% mortality) and concluded that the procedure was unlikely to significantly increase risk relative to a traditional approach. However, Gologorsky et al. (2011) noted a high rate of postoperative delirium and ventricular wall violation that they concluded may increase the risk of adverse neurological sequelae. Both of these studies were based on retrospective chart review of relatively small samples sizes (156 and 81 PD DBS patients, respectively), and there has not been a direct comparison of side effects of a unilateral vs. *trans-*ventricular approach within a controlled study.

Finally, a single retrospective study of 94 patients experiencing hardware complication at a hospital in Japan focused on surgical methods to reduce hardware extrusion, which was noted to be a significant problem within their center (12.8%, split equally between extrusion at the scalp and extrusion at the battery). The authors suggested the use of tension-free, well-vascularized locoregional flaps as opposed to primary closure to reduce scalp extrusion and primary closure and repositioning in a new surgical bed to correct IPG extrusion (Zhang et al., 2022).

The studies presented above highlight to potential utility of several interesting methods, but further research is needed to compare different techniques, and there are many techniques and technologies that have not specifically explored rates of complication at all, so far as the authors are aware. While robotic-assisted stereotaxy, interventional MRI-guided procedures, and different electrode placements (e.g., STN vs. GPi vs. new targets such as PPN or SN) are all rapidly evolving and of considerable interest in terms of patient care both within the hospital and following discharge, no resultant articles focused specifically on complications or hospitalizations post-operatively were found that discuss differences based on these factors.

## 7. Limitations

While existing literature has found that DBS demonstrates a relatively safe profile, especially given its proven record of significant improvement in PD symptoms, interpretability is affected by the fact that most studies examining these DBS complications thus far have been retrospective or observational cohorts. Thus, it is difficult to determine whether increased hospitalizations and complications following DBS are a result of the DBS surgery itself or increased patient risk for these complications, either due to advanced disease or due to other factors predisposing them to seek out DBS whereas others choose not to pursue surgery. Few papers have had a control group of patients not receiving DBS that can be assumed to be comparable to the DBS group. Because DBS is an FDA-approved and widely accepted and practiced procedure, it is unlikely that any kind of randomized study will occur. However, large, multi-site, longitudinal databases to trace PD patients through time provide the possibility for stratification and analysis separating the effects of preoperative factors from surgical outcomes, especially with the increasing use of artificial intelligence. Several of the recent large-scale retrospective papers highlighted within the long-term effects section of this paper show the early promise of such research.

More research is also needed to directly compare different surgical approaches to DBS, which may be done in a randomized head-to-head fashion. There are any number of potential approaches for anesthesia, lead trajectory, determination of proper lead placement (based on MER and/or intraoperative imaging techniques), and stabilization/fixation. Surgeon preference, often based on individual training/experience, has played a large role in determining which methods are used at different centers, but systematic comparison would be enlightening.

The role of target selection, especially between STN and GPi, and the resultant rates of hospitalization and side effects, is also an incredibly important avenue of research for the future. While the comparative effect on motor symptoms between the two targets seems well-balanced, differences in effect on hospitalization or complications within the hospital could make one site more advisable for certain individuals with pre-operative concerns from those symptoms. The authors did not find any articles directly comparing hospitalization or complication rates for STN vs. GPI; however, some papers analyzed relative rates of dysphagia and falls. While results have been somewhat conflicting, it would appear that GPi DBS may better spare swallowing ability (Troche et al., 2014; Yu et al., 2020; Henry et al., 2022) and gait and balance (Celiker et al., 2019), likely having a neutral to beneficial effect while STN DBS may lead to degradation in these symptoms in at least some cases.

Additionally, it should be reiterated that, in order to ensure a cohesive review within reasonable length, this review focused largely on hospital-related and severe complications following DBS. However, even in the absence of such events, PD symptoms and quality of life for patients can be significantly affected in both positive and negative ways. The positive effects of DBS on most symptoms of PD are well-known and were briefly highlighted in the introduction to this paper. However, there are also risks of decline in certain aspects of functioning, especially cognition, following DBS, which are well-researched and discussed in other articles (Parsons et al., 2006; Rothlind et al., 2015, 2022, Tröster et al., 2017).

## 8. Conclusion and future research

Parkinson’s Disease (PD) is a neurodegenerative disease without a cure currently and is known to be associated with increased hospitalizations as well as increased risks of various complications while hospitalized. Deep brain stimulation (DBS) is an increasingly utilized intervention for those living with advanced PD. However, while DBS has been shown to be a safe and efficacious treatment for PD, even a low rate of complications may present a significant source of further disability and increased health care costs for those affected. The presence of DBS, especially in the early post-operative period where wounds still need to be checked and programming settings are still changing, may also significantly affect the care patients need within the hospital. Thus, it is important to understand the sources and risk factors related to adverse surgical outcomes to ensure that treatments are well-considered and targeted to provide the most benefit to individual patients. Furthermore, with the growing number of aging PD patients who have undergone DBS, it is important to understand how this intervention may impact hospitalizations and associated care in this group.

This review characterizes the current literature regarding hospitalization associated risks and morbidity in DBS patients with PD. Perioperative outcomes and complications related to the initial implantation, particularly mortality, delirium, length of stay, and discharge disposition seem to be the more well-assessed variables. Some aspects of surgical technique have been assessed, though head to head comparisons are limited, with the exception of those related to type of anesthesia. The studies are primarily retrospective and heterogeneous. More information is needed to really understand how the size of the operative center, the experience of the surgeon, patient and site selection, and operative technique and technology play a role in the differing results. There has also not been systematic comparison how rates of DBS complications differ with time, and whether advancements in technology have lowered risk in recent years.

In terms of long term outcomes, the conclusions are less clear. There does seem to be a small group of DBS patients who require readmission for device related issues including infection, extrusion, hardware failure, etc., implying a generally higher risk of hospitalizations in those who undergo DBS. When DBS related hospitalizations were excluded in one analysis, the rate of hospitalization over time was seemingly similar to those without DBS. Other analyses not included in this review that generally assessed the risk of hospitalization and rehospitalization in PD patients have found DBS to be a risk factor for recurrent inpatient encounters (Shahgholi et al., 2017). Overall, however, follow-up durations even in the “long” term studies were relatively short with only rare studies following patients for more than a year after DBS placement.

It is also important to note that none of the studies analyzed in this review compared hospitalization results for those patients admitted post-operatively to hospitals that perform DBS and thus likely have more experience with patient populations with PD vs. those admitted to community hospitals without such dedicated resources. Many articles, both within this special issue and elsewhere, have noted that some hospitals may be inexperienced with PD-specific aspects of care, such as the importance of medication timing, the screening and management of dysphagia to reduce incidence of aspiration pneumonia, ensuring sufficient exercise to maintain motor abilities without allowing increased falls, and avoiding contra-indicated medications (Palmer et al., 2021; Brooks, 2023; Goldin et al., 2023; Shurer et al., 2023). It is unclear if perhaps having DBS may even improve outcomes in small community hospitals where there may be less awareness regarding medication timing and access in PD, both of which are known risks for prolonged hospitalization in PD patients. Education in these things may dramatically improve patient experiences and outcomes, but the best ways to distribute this education are still uncertain.

Some aspects of hospital care may differ specifically related to DBS as well, further increasing the need for education and collaboration between providers. For example, effects of delayed or missed medication doses may not be as immediately apparent for individuals with DBS, as fluctuations and dyskinesias are less severe, but it is still key that medications be kept as stable as possible. Additionally, rehabilitation may be immensely helpful in the post-operative period to improve DBS outcomes (Allert et al., 2018; Sato et al., 2022). Rehabilitation is also easily adjusted and may be targeted to specifically address areas of concern such as swallowing or balance or may be provided based on signs of decline to prevent administrative burden of providing therapy to individuals likely to recover without incident on their own.

It is unclear what role DBS may play, if any, in the long term sequelae of PD progression as it relates to hospitalizations. Is there a difference in mortality? Rates of hospital admissions for problems of advanced disease such as pneumonia and falls? Do those who are admitted for problems unrelated to the DBS itself have different lengths of stay, inpatient complication rates, and discharge dispositions? There is an aspect of phenotypic variation, i.e., those who were DBS candidates and those who were not, that will be a confounder in these analyses, but currently, there is very limited knowledge regarding long term outcomes and prospective, multicenter, outcomes studies will be of considerable value. The only literature providing insight comes from the prospective NPF study which suggests that over time, DBS patients may have higher rates of hospitalizations (Hassan et al., 2013).

It should be noted that while some analyses have looked at socioeconomic status, insurance type, rural v. urban centers, and academic v. non-academic centers as variables in outcomes, race/ethnicity and sex have not been specifically investigated. It is already established that DBS is underutilized in racially marginalized groups in the US, and continues to be an issue but even less is known about surgery related inpatient complications and hospitalization associated aspects in those who do undergo the procedure (Cramer et al., 2022).

In conclusion, there are risks associated with DBS surgery itself, particularly related to the procedure and within the 90-day period following the initial lead placement. There are also risks associated with hospitalization, which are increased in patients with PD, and may be further aggravated by hospitalizations, both planned and unplanned, resulting from DBS. DBS patients may also have specific care needs both due to advancing disease and DBS-related programming and wound care. More studies are needed to better understand DBS as a variable over time for PD patients as it pertains to hospitalizations and how education and rehabilitation programs may improve outcomes of hospitalizations for this at-risk patient population.

## Data availability statement

The original contributions presented in this study are included in this article/supplementary material, further inquiries can be directed to the corresponding author.

## Author contributions

MO: Conceptualization, Data curation, Methodology, Writing—original draft, Writing—review and editing. HS: Writing—review and editing. FP: Writing—review and editing. SA: Conceptualization, Data curation, Methodology, Writing—original draft, Writing—review and editing.
